# Viruses in neurodegenerative diseases: More than just suspects in crimes

**DOI:** 10.1371/journal.ppat.1010670

**Published:** 2022-08-04

**Authors:** Pascal Leblanc, Ina Maja Vorberg

**Affiliations:** 1 Institut NeuroMyoGène INMG-PGNM, Physiopathologie et Génétique du Neurone et du Muscle, UMR5261, Inserm U1315, Université Claude Bernard UCBL-Lyon1, Faculté de Médecine Rockefeller, Lyon, France; 2 German Center for Neurodegenerative Diseases Bonn (DZNE), Bonn, Germany; 3 Rheinische Friedrich-Wilhelms-Universität Bonn, Bonn, Germany; National Institutes of Health, UNITED STATES

## Abstract

Neurodegenerative diseases (NDs) such as Alzheimer’s and Parkinson’s disease are fatal neurological diseases that can be of idiopathic, genetic, or even infectious origin, as in the case of transmissible spongiform encephalopathies. The etiological factors that lead to neurodegeneration remain unknown but likely involve a combination of aging, genetic risk factors, and environmental stressors. Accumulating evidence hints at an association of viruses with neurodegenerative disorders and suggests that virus-induced neuroinflammation and perturbation of neuronal protein quality control can be involved in the early steps of disease development. In this review, we focus on emerging evidence for a correlation between NDs and viral infection and discuss how viral manipulations of cellular processes can affect the formation and dissemination of disease-associated protein aggregates.

## Evidence for an association of viral infections with neurodegenerative diseases

Neurodegenerative diseases (NDs) are fatal chronic diseases of the central nervous system (CNS), including Alzheimer’s disease (AD), Parkinson’s disease (PD), amyotrophic lateral sclerosis (ALS), and transmissible spongiform encephalopathies (TSEs). A hallmark of NDs is the intra- or extracellular deposition of cellular proteins into ordered high-molecular weight fibrils, termed amyloid. Protein aggregation follows a nucleated polymerization process, in which misfolded proteins spontaneously coassemble into oligomers (nucleation) that reorganize into beta-sheet-rich fibrils. Amyloid fibrils then act as seeds that bind and convert proteins of the same kind into their abnormal isoforms (seeding). Protein aggregation occurs sequentially in anatomically connected areas, suggesting a progressive spreading throughout the CNS of affected individuals [1]. Approximately 90% of NDs occur sporadically, and only few cases are linked to mutations in aggregation-prone proteins or proteins involved in their processing or trafficking. The etiology of idiopathic NDs remains unknown. NDs are multifactorial diseases, triggered by enhanced age as well as genetic and environmental risk factors. Pathogens, and especially viruses, are suspected to act as etiological factors in several NDs. An impressive number of studies highlights that viruses, through their capacity to hijack the host cell machinery and induce inflammation, trigger and/or contribute to degenerative processes. Viral infections can activate astrocytes and microglia or induce CNS infiltration by peripheral immune cells, thereby causing neuroinflammation (reviewed in [2]). Some viruses can enter the CNS and affect neurodegeneration via lytic egress from infected neurons by impairing neuronal processes or by inducing neuronal apoptosis. In this review, we discuss how viruses can also directly contribute to disease-associated protein misfolding and subsequent processes of protein aggregate spreading.

AD affects 40 million people worldwide and is associated with extracellular deposition of Aβ amyloid as plaques and the intracellular deposition of hyperphosphorylated Tau protein as neurofibrillary tangles. Production of Aβ amyloid is a critical initial event in disease progression, but what exactly triggers Aβ fibrillization in idiopathic AD is unknown. Epidemiological and experimental evidence suggest that infection with or reactivation of herpesviruses can increase the risk of developing AD or AD-like pathology (reviewed in [3]). Herpesviruses are neurotropic viruses that establish lifelong latent infections in sensory neurons. Herpes simplex virus-1 (HSV-1) periodically reactivates and can thereby infiltrate the brain and cause encephalitis or establish CNS latency. Several studies link HSV-1 to AD. Indeed, HSV-1 seropositivity appears to increase the risk for developing AD [4] and HSV-1 DNA can be detected in Aβ plaques [5]. In cellular models and mice, repeated reactivation of HSV-1 infection results in a progressive accumulation of AD biomarkers Aβ and hyperphosphorylated Tau (reviewed in [3]). Also, aged patients infected with human immunodeficiency virus type 1 (HIV-1) and treated with highly active antiretroviral therapy suffer from neurocognitive disorder associated with the deposition of Aβ and hyperphosphorylated Tau and could thus be at greater risk for developing AD-like disorder (reviewed in [6]).

PD, the second most common ND, is characterized by the degeneration of dopaminergic neurons in the substantia nigra and the accumulation of α-Synuclein as Lewy bodies in neurons. Influenza-A, flaviviruses, and herpesviruses can induce acute or chronic Parkinson-like symptoms or post-encephalitic parkinsonism (reviewed in [7,8]). Retrospective cohort studies reported an increased risk for developing PD after infection with hepatitis C and B viruses (HCV and HBV) [9]. Experimentally, mice infected with neurotropic influenza-A virus exhibit α-Synuclein inclusions in dopaminergic neurons as well as inflammatory processes and microglial activation [10].

ALS is a motor neuron disease that affects nerves in brain and spinal cord. Cytoplasmic mislocalization and accumulation of RNA-binding proteins such as TDP-43 or FUS are hallmarks of ALS and a subset of frontotemporal dementia. ALS is accompanied by the up-regulation of a human endogenous retrovirus (HERV-K), a normally epigenetically silenced and replication-incompetent remnant of ancient germline infection [11]. The presence of enteroviruses in brains and cerebrospinal fluid of ALS patients is debated (reviewed in [12]). However, in mice, infection with 2 enteroviruses induced TDP-43 accumulation and sustained inflammation [13]. Infection of mice with the picornavirus Theiler’s murine encephalitis virus (TMEV) caused an ALS-like phenotype with cytoplasmic inclusions of TDP-43 and FUS in motor neurons and glial cells [14].

TSEs are a special class of neurodegenerative disorders that are caused by “proteinaceous infectious particles” (prions) that consist predominately, if not entirely, of misfolded prion protein PrP (PrP^Sc^; “Sc” for scrapie) [15]. The protein-only hypothesis for prions is now widely accepted by the scientific community and supported by a wealth of in vitro and in vivo evidence. Nevertheless, early investigations identified virus-like particles and tubulovesicular structures in natural and experimentally induced TSEs and in a prion-infected cell line [16,17]. In sheep, infection with Maedi visna virus–induced mastitis and increased transmission of prions to suckling lambs [18]. In a cellular model, infection with a ruminant lentivirus enhanced infectious PrP^Sc^ accumulation [19]. A recent study in vitro reported that infection of neuroblastoma cells with neurotropic influenza-A H1N1 virus even triggered the spontaneous formation of PrP^Sc^, which was subsequently shown to be infectious to mice [20]. In summary, epidemiological and experimental evidence links viral infections to the development of NDs and suggests that viruses have the potential to directly modulate processes that lead to protein aggregation.

## Viral perturbations of cellular functions affect protein aggregation

**Fig 1:**
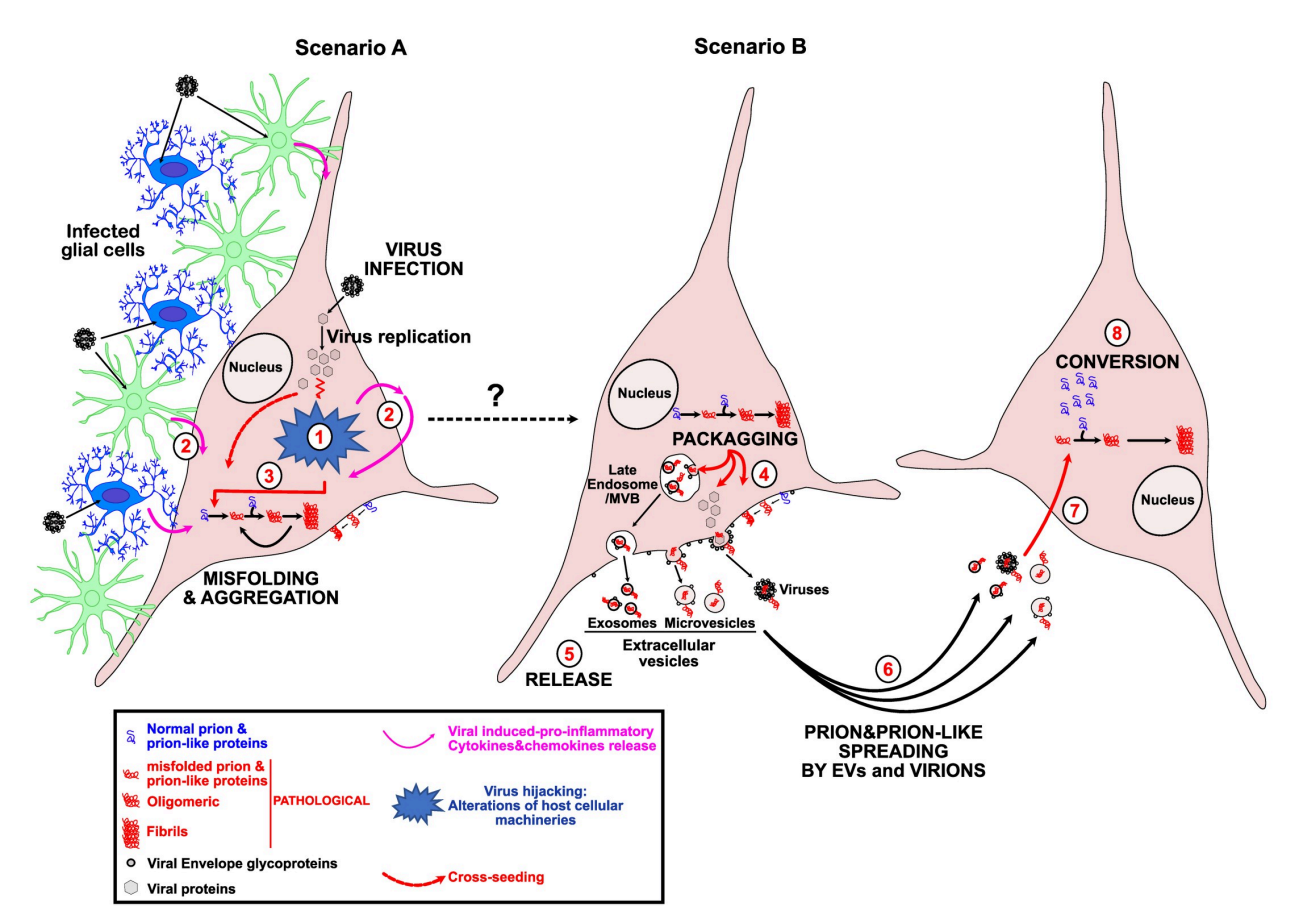
Virus infection and step-by-step misfolding and aggregation, spreading, and prion-like conversion. **Scenario A**: Viruses hijack the host cellular machineries and pathways for their own benefit to efficiently drive their replication. To this end, viruses developed different strategies to impair intracellular trafficking like nucleocytoplasmic export, the endo-lysosomal secretory and degradation pathways as well as all the machineries involved in protein quality control and in proteostasis (1). In response to infection, infected host cells, including glial cells, reply through the expression of restriction factors and secretion of proinflammatory cytokines and chemokines (2). Inactivation of protein quality control and induction of neuroinflammation are major events leading to the misfolding and aggregation processes of ND-associated proteins like PrP, α-Synuclein, APP/Aβ, Tau, TDP-43, and FUS (3). **Scenario B**: Misfolded pathological proteins can have different etiology. They can be induced by aging, by mutations in susceptibility genes or ND-associated genes themselves, or by environmental stressors such as pathogens-like viruses (see dotted black line) or repeated contacts with chemicals. In virus-infected cells, misfolded proteins can be secreted in EVs that bud from the plasma membrane (microvesicles and viral particles) or from the surrounding membrane of MVBs (exosomes) (4). Like viral particles, EVs are released (5) in presence or absence of viral Envelope glycoprotein at their surface, conferring broadened cell tropism and increased endosomal escape essential for efficient spreading (5, 6). Viral particles and EVs containing pathological protein aggregates enter target cells through different mechanisms, including the one mediated by the interaction between the viral Envelope glycoprotein and its host membrane receptor (7). Once introduced into the target cells, protein aggregates induce the conversion of their normal counterpart into aggregated isoforms through a conformational templating mechanism (8). EV, extracellular vesicle; MVB, multivesicular body; ND, neurodegenerative disease.

Viruses are obligate parasites that hijack host cellular machineries and pathways for successful infection and replication (**Fig 1**). Virtually, all cellular processes, including intracellular trafficking and cytoskeleton dynamics, become altered during viral infection and redirect resources and energy flow toward efficient viral replication. To maintain proteome integrity and cellular health, concentration, proper folding, activity and localization of proteins need to be tightly controlled at levels of translation, posttranslational modification, and degradation. Chaperones, the proteasome, and the autophagy-endo-lysosomal pathway maintain the integrity of the so-called proteostasis. Unfortunately, both capacity and efficiency of protein quality control decline during aging and upon environmental stressors. Viral infections profoundly alter proteostasis, rendering cells more vulnerable to protein misfolding. Additionally, antiviral cell responses like the secretion of proinflammatory cytokines and chemokines can contribute to protein misfolding and aggregation. In genetic forms of NDs, this can exacerbate preexisting defects and can decrease the time to disease onset [21,22]. Here, we discuss briefly several mechanisms how viral infection can contribute to or elicit protein misfolding and aggregation at a cellular level. We apologize that we cannot cover all studies on this subject. For more in-depth information also in terms of inflammation, the reader is referred to the excellent reviews cited within this article.

One way how viruses can affect protein aggregation is by increasing the expression of host-encoded aggregation-prone proteins, as has been demonstrated for virally induced enhanced expression of α-Synuclein, TDP-43, FUS, Aβ, or PrP (**Table 1**). PrP^C^ expression is up-regulated upon infection with adenovirus, HCV, or retroviruses such as HIV-1 and murine leukemia virus in cell culture or in vivo [23–26]. α-Synuclein is up-regulated upon West Nile virus (WNV) [27] or coxsackievirus B3 (CVB3) infection [28], while levels of TDP-43 and FUS increase upon herpesvirus infection [29]. Some ND-associated proteins become up-regulated upon viral infection to participate in first-line defense against pathogens (reviewed in [8]). A risk of increased expression is that high concentrations of aggregation-prone proteins lower the nucleation barrier for protein aggregation and can result in amyloid formation (reviewed in [8]).

**Table 1:** Effect of viral infection on the expression and aggregation of ND-associated proteins.

prion/prion-like Viruses	α-Synuclein	Tau	APP/Aβ	TDP-43	FUS	PrP
**Retrovirus**						
**HIV-1**	•[30]	•[31]	•[32]	••[33]	•[21]	•[34] •[25]
**CAEV**						•[19]
**IMERV-1**						•[26]
**HTLV-I**		•[35]				
**Herpes virus**						
**HSV1**		•[36]	•[36]			
				•[29]	•[29]	
**HSV2**		•[37]	•[37]			
**CMV**		•[38]	•[38]			
**VZV**			•[39]			
**HHV6/7**		•[40, 41]	•[40, 41]			
**Flavivirus**						
**HCV**						•[42]
**WNV**	•[27]					
**Enterovirus/Picornavirus**						
**CVB3**	••[28]	•[43]		•[13, 43]		
**Ev71**				•[44]		
**TMEV**				•[14]	•[14]	
**RBV**					•[21]	
**Influenza virus**						
**H1N1**	•[45]					•[20]
**H5N1**	•[10]					
**Metapneumovirus**						
**RSV**					•[22]	
**Adenovirus**						
**Adenovirus 5**						•[23]

UPREGULATION •/AGGREGATION•

CAEV, caprine arthritis encephalitis virus; CMV, cytomegalovirus; CVB3, coxsackiervirus B3; Ev71, enterovirus 71; HCV, hepatitis C virus; HHV6/7, human herpes virus 6/7; HIV-1, human immunodeficiency virus type 1; HSV-1/2, herpes simplex virus 1; HTLV-I, human T lymphotropic virus type I; H1N1/H5N1, human neurotropic influenza type A virus; IMERV-1, immune activated murine endogensous retrovirus 1; ND, neurodegenerative disease; RBV, rabies virus; RSV, respiratory syncytial virus; TMV, Theiler encephalomyelitis virus; VZV, varicella zoster virus; WNV, West Nile virus.

Viral infection can also indirectly affect protein misfolding and aggregation by up-regulating or activating proteins that mediate the posttranslational processing or clearance of aggregation-prone proteins (**Fig 1**). For example, HSV-1 infection activates the Glycogen synthase kinase GSK3β, which hyperphosphorylates Tau and promotes built-up of Aβ (reviewed in [8]). Viral proteins can also reduce the activity of enzymes involved in degradation of disease-associated proteins, such as HIV-1 Tat, which reduces the activity of the neuronal endopeptidase Neprilysin involved in Aβ clearance [32]. Also, proinflammatory cytokines released by microglia can affect expression and processing of ND-associated proteins (reviewed in [2]).

Viral infections can also alter the cellular fate of disease-associated proteins. Virus-induced dysregulation of intracellular trafficking between nucleus and cytosol or of the secretory pathway and the endo-lysosomal system can increase local concentrations of ND-associated proteins. This, in turn, lowers the thermodynamic barrier to spontaneous aggregation, provides a cellular environment prone for aggregation, or impairs clearance of misfolded proteins (**Fig 1**). An example is the HIV-1 protein Tat that which redirects the amyloid precursor protein APP to lipid rafts in the membrane, a region that also contains the enzymatic machinery that produces Aβ from its precursor [46]. Further, infection of cells or mice with enteroviruses or picornavirus TMEV induced the cytoplasmic aggregation of TDP-43 and/or FUS due to a compromised nuclear import mediated by viral proteins [13,14,43]. Thus, virally induced changes in intracellular trafficking can have profound consequences for protein aggregation.

Protein aggregates can be detrimental to the cell and are subject to clearance by autophagy, a process that mediates the degradation of cytosolic components. Impaired autophagy is a characteristic of aging and NDs (reviewed in [8]). Neurons are especially vulnerable to autophagy impairment. Autophagy is also a crucial player in antiviral defense, as it controls viral replication as well as antiviral innate and acquired immune responses (reviewed in [47]). Consequently, viruses have evolved strategies to manipulate or even exploit autophagy for their own benefits. Virally induced dysregulation of autophagy can have detrimental effects on the degradation of disease-associated proteins. For example, HHV-6A (human herpes virus 6A) or HSV-1 infections of astrocytoma or neuronal cells reduced the autophagic flux (the degradation capacity of autophagy), subsequently resulting in increased Aβ production and Tau hyperphosphorylation [40,41,48]. Similarly, infection of mice with CVB3 significantly inhibited the late steps of autophagic process through the catalytic activity of the viral proteinase 3C that disrupts the SNARE complex, which is involved in autophagosome–lysosome fusion. This resulted in perinuclear clusters of organelles and autophagy-related structures colocalized with phosphorylated α-Synuclein aggregates [28]. In a dopaminergic neuron-like cell line and immunocompromised mice, neurotropic H1N1 and H1N5 influenza-A virus infections induced α-Synuclein aggregates, likely through impaired autophagosome formation and subsequent inhibition of the autophagic flux [10,45]. Severe Acute Respiratory Syndrome Coronavirus 2 (SARS-CoV-2) infection sometimes causes neurological, psychiatric, and AD-like symptoms even after acute infection subsides, a stage termed long COVID [49]. The impact of SARS-CoV-2 infection and associated inflammation on the deposition of ND-associated proteins is not fully investigated at the present time. However, recent data revealed that a SARS-CoV-2 infection can directly or indirectly promote the hyperphosphorylation of Tau. Interestingly, SARS-CoV-2 infection and viral proteins ORF3a, ORF7a, M or NSP6 can impair the autophagy process by blocking the fusion of autophagosomes and related structures with lysosomes [49]. Strikingly, SARS-CoV-2 Nucleocapsid also associated with TDP-43 or FUS proteins in liquid–liquid phase separated assemblies [50]. Further in-depth characterization is necessary to reveal the impact of SARS-CoV-2 infection on neurodegenerative processes. In summary, virally induced dysregulation of autophagy could act as an initial trigger of protein misfolding or exacerbate already ongoing protein aggregation (**Fig 1**).

## Viral gene products can directly nucleate amyloid formation

Amyloid formation follows a process of nucleated polymerization, which is strongly enhanced by binding of monomers to certain surfaces that might act as scaffolds that increase local concentrations of ND-associated proteins, such as nucleic acids or glycosaminoglycans (reviewed in [8]). Intriguingly, also herpesviruses can drastically increase the fibrillization of Aβ peptide and may accelerate the progression of AD [51]. Interestingly, enhanced Aβ fibrillization and accumulation was also found to play a protective role in CNS innate immunity. Aβ may act as an antimicrobial peptide or restriction factor, an activity also described for ND-associated proteins such as α-Synuclein, TDP-43, FUS, or PrP^C^, which become up-regulated during certain viral infections [8,51]. Short stretches of sequence homology between viral proteins and ND-associated proteins could mediate interactions that promote fibrillization, a process called cross-seeding (reviewed in [52]) (**Fig 1**). Interestingly, viral proteins such as HSV-1 glycoprotein K or HIV-1 gp120 contain short stretches of aggregation-prone regions with sequence homology to regions in Aβ, suggesting that they could bind to Aβ and drive its fibrillization (reviewed in [52]).

## Viral proteins can increase the spreading of disease-associated protein aggregates

Viruses are obligate intracellular parasites that enter cells by binding to specific receptors on the host cell surface. Contact between enveloped viruses and the cell membrane is mediated by viral glycoproteins that can exhibit fusogenic activity, enabling efficient release of viral capsids into the cytosol. For dissemination, viruses also transmit viral genomes and/or proteins by direct cell contact or by extracellular vesicles (EVs), including microvesicles or exosomes (**Fig 1,** scenario B) [53]. Both cell contact and uptake of EVs with viral cargo is mediated by viral Envelope glycoproteins. Viral glycoproteins can also affect the intercellular dissemination of disease-related protein aggregates that can be transmitted to other cells via cell contact or when packaged actively or passively into EVs. Expression of Envelope glycoprotein VSV-G of vesicular stomatitis virus or SARS-CoV-2 spike S by cells containing Tau aggregates enhanced spreading of Tau misfolding to naïve cells, either by close cell contact or EVs [54]. Further analysis revealed that aggregated Tau was recruited to EVs. The effect of viral glycoproteins on the prion-like spreading of protein aggregation was not restricted to Tau, as also artificial cytosolic protein aggregates composed of a yeast prion protein domain as well as PrP^Sc^ could be efficiently transmitted to other cells by Envelope-covered EVs. Experiments with VSV-G glycoprotein mutants demonstrated that the fusogenic activity of the viral protein was a main driver of intercellular protein aggregate induction. A low pH in the early endosomal pathway was required to trigger VSV-G activation and subsequent release of protein aggregate seeds into the cytosol, where aggregation of homotypic proteins was initiated.

Spreading of pathogenic protein aggregates can also be enhanced in cells coinfected with murine leukemia virus (MuLV) and mouse-adapted prion disease (**Fig 1**) [55]. In contrast to other ND-related proteins, PrP^Sc^ is tethered to the cell surface and to EVs of infected cells by a glycosylphosphatidylinositol-anchor. Coinfection with MuLV and prions resulted in an enhanced release of prion infectivity. Detailed analysis demonstrated that PrP^Sc^ was recruited to EVs that transmitted prion infectivity to recipient cells. Remarkably, prion infectivity also associated with retroviral fractions, which induced prion infection in naïve cells. These results suggest that both EVs and virions decorated with PrP^Sc^ can serve as vehicles for prion transmission. Retroviral polyprotein Gag, coding for Nucleocapsid, Matrix, and Capsid, was identified as a main driver of virion and EV release. Consistent with this, Gag expression also increased sustained infection of cells with chronic wasting disease prions [56]. Thus, at least in cellular models, viral proteins can directly modulate the intercellular dissemination of protein misfolding, either by increasing EV release or by catalyzing membrane attachment and fusion with target cells required for protein aggregate transfer. As viruses actively dysregulate EV biogenesis and secretion, we anticipate general effects of viral infection on intercellular aggregate transmission [53].

## Antivirals as therapeutics to treat NDs

As aberrant deposition of Aβ is believed to initiate the detrimental cascade of Tau aggregation and neuronal death [57], current clinical trials for AD mainly target Aβ, albeit with limited therapeutic outcome [58]. Antivirals might thus represent interesting alternative drug candidates for the treatment of NDs. In cell culture, antivirals were able to lower HSV-1-induced Aβ production and phosphorylated Tau [59] or influenza-A-mediated α-Synuclein aggregation [45]. Analyses of large medico-administrative databases support this and suggest that antiherpetic therapy could decrease the risk of developing AD [60]. However, a Phase II pilot trial with a high dose oral administration of antiherpetic drug valacyclovir for 4 weeks failed to show changes in ND markers in patients with early-stage AD [61]. Another meta-analysis of cohort and case–control studies recently suggested that antiviral treatment for HCV could reduce the risk for developing PD [9]. Antiviral therapy is currently also assessed for treatment of ALS. Antiretroviral combination therapy lowers transcript levels of HERV-K subtype HML-2, an endogenous retrovirus family reported to be derepressed in ALS [62]. A Phase IIa clinical trial conducted with ALS patients confirmed that antiretroviral therapy (with efficacy against HERV-K HML-2) showed a trend toward delayed disease progression in patients with virological response. While this result is encouraging, randomized controlled trials are now warranted to assess potential positive outcomes on NDs.

Clearly, much has to be learned on the role of viral infections in the onset and/or progression of NDs. In case a direct cause-correlation can be established, are single infections, multiple reinfections, or recurrent reactivations of latent viruses responsible for the deadly cascade of neuroinflammation, neuronal injury, and protein aggregation? In terms of antiviral therapy, an important problem that would need to be solved is when to initiate treatment and if short- or long-time antiviral interventions are required to slow disease progression. Of note, also other microbes have been implicated in the onset of certain NDs, as have several toxins or chemicals. Thus, different environmental factors could trigger the same cellular processes that culminate in a fatal cascade of neurodegeneration. A better understanding of how environmental stressors such as viruses trigger neurodegenerative processes will open up new avenues for disease interventions.
